# Supplementary material for: Viruses of sulfur oxidizing phototrophs encode genes for pigment, carbon, and sulfur metabolisms
Source: Commun Earth Environ. 2023 Apr 19;4(1):126. doi: 10.1038/s43247-023-00796-4 (PMC11041744; doi:10.1038/s43247-023-00796-4)
Supplement: Supplementary file 1 — Description of Additional Supplementary Files [file 43247_2023_796_MOESM1_ESM.pdf]

Description of Additional Supplementary Files

File Name: Supplementary Data 1

Description: Access codes for purple and green sulfur bacteria genomes retrieved from the National Center for Biotechnology Information (NCBI) RefSeq.

File Name: Supplementary Data 2

Description: List of predicted phage-hosts pairs, phage genome quality, and phage AMGs.
