# Supplementary material for: Viruses of sulfur oxidizing phototrophs encode genes for pigment, carbon, and sulfur metabolisms
Source: Commun Earth Environ. 2023 Apr 19;4(1):126. doi: 10.1038/s43247-023-00796-4 (PMC11041744; doi:10.1038/s43247-023-00796-4)
Supplement: Supplementary file 2 — Supplementary Information [file 43247_2023_796_MOESM2_ESM.pdf]

Supplementary Figures and Tables: Viruses of sulfur oxidizing phototrophs encode genes for pigment, carbon, and sulfur metabolisms

Poppy J. Hesketh-Best[1*], Alice Bosco-Santos[2*], Sofia L. Garcia[1], Molly D. O'Beirne[3], Josef P. Werne[3], William P. Gilhooly III[4], Cynthia B. Silveira[1]

[1] Department of Biology, University of Miami, Coral Gables, USA.

[2] Institute of Earth Surface Dynamics, University of Lausanne, Lausanne, Switzerland.

[3] Department of Geology & Environmental Science, University of Pittsburgh, Pittsburgh, USA.

[4] Department of Earth Sciences, Indiana University-Purdue University Indianapolis, Indianapolis, USA.

[*] **These authors contributed equally.**

[#]**Correspondence to:** cynthiasilveira@miami.edu

Keywords: bacteriophages, purple sulfur bacteria, green sulfur bacteria, primary production, auxiliary metabolic genes, biosignatures

Supplementary Table 1. Summary of the data utilized and generated in this study.

	**Chromatiaceae**	**Ectothirhodospirales**	**Chlorobiota**	**Lime Blue**	**Poison Lake**
Data type	NCBI whole and draft genomes	NCBI whole and draft genomes	NCBI whole and draft genomes	Sediment cellular metagenome	Freshwater cellular metagenome
#genomes	98	115	33	N/A	N/A
#reads	N/A	N/A	N/A	3,903,732	19,479,434
#high quality reads	N/A	N/A	N/A	154,123	1,166,886
#contigs assembled	N/A	N/A	N/A	21,893	2,965
#phyla (kraken2 on reads)	N/A	N/A	N/A	44	55
#family (kraken2 on reads)	N/A	N/A	N/A	356	481
#genera (kraken2 on reads)	N/A	N/A	N/A	927	1840
#phages predicted (VIBRANT)	123	111	17	2,742	5,809
#high quality MAGs	N/A	N/A	N/A	17	10
#predicted AMGs	35	25	11	544	52
#AMGS of interest	5	1	1	50	9

Supplementary Table 2. Taxonomy and quality of MAGs. Quality was estimated by CheckM and taxonomy by GTBD-Tk. * denotes potential PSB MAGs, and † denotes a MAG identified as a potential host to a phage by CRISPR spacers. (Comp: bin completeness; Contam: bin contamination).

	**Bin**	**Binner**	**Phylum**	**Lowest taxonomic classification (GTDB-tK)**	**Comp.**	**Contam.**	**Relative Abundance (%)**	**dRep Cluster**
**PL**	PL_nanophase_bin.20*	NanoPhase	Proteobacteria	s__Thiohalocapsa sp001469165	58.86	0.7	22.96	cluster10
**PL**	PL_lrbinner_bin-0*	LRBinner	Proteobacteria	s__Thiohalocapsa sp001469165	88.5	2.49	18.26	cluster10
**PL**	PL.bin01*†	CONCOCT+ MaxBin2+ MetaBAT2	Proteobacteria	s__Thiohalocapsa sp001469165	85.17	1.17	17.05	cluster10
**PL**	PL.bin04†	CONCOCT+ MaxBin2+ MetaBAT2	Desulfobacterota_I	g__Desulfonatronum	89.53	0.6	6.12	cluster13
**LB**	LB_nanophase_bin.80	NanoPhase	Chloroflexota	g__CAIMUM01	52.15	0.2	5.90	cluster21
**PL**	PL.bin03	CONCOCT+ MaxBin2+ MetaBAT2	Deinococcota	g__JAABTL01	76.11	2.97	4.37	cluster11
**LB**	PL.bin05	CONCOCT+ MaxBin2+ MetaBAT2	Bacteroidota	f__4484-276	82.29	3.42	4.08	cluster9
**PL**	PL_nanophase_bin.15	NanoPhase	Bacteroidota	f__4484-276	57.98	1.08	3.08	cluster9
**PL**	PL.bin02	CONCOCT+ MaxBin2+ MetaBAT2	Verrucomicrobiota	o__UBA8416	76.74	3.05	2.02	cluster1
**PL**	PL_nanophase_bin.17	NanoPhase	Verrucomicrobiota	o__UBA8416	73.87	1.46	1.97	cluster1
**PL**	PL.bin06	CONCOCT+ MaxBin2+ MetaBAT2	Chloroflexota	f__A4b	68.8	0	1.92	cluster8
**LB**	LB_nanophase_bin.5	NanoPhase	Cyanobacteria	g__Cyanobium	77.11	1.45	1.14	cluster19
**LB**	LB_nanophase_bin.226	NanoPhase	AABM5-125-24	g__JAHIZR01	69.55	4.5	0.74	cluster18
**LB**	LB_nanophase_bin.11	NanoPhase	Patescibacteria	f__GWA2-36-10	62.23	0	0.67	cluster14
**LB**	LB.bin03†	CONCOCT+ MaxBin2+ MetaBAT2	Patescibacteria	f__GWA2-36-10	62.23	0	0.61	cluster14
**LB**	LB.bin05	CONCOCT+ MaxBin2+	Planctomycetota	g__UBA7708	70.8	4.65	0.57	cluster15

	Bin	Binner	Phylum	Lowest taxonomic classification (GTDB-tK)	Comp.	Contam.	Relative Abundance (%)	dRep Cluster
		MetaBAT2						
**LB**	LB.bin07	CONCOCT+ MaxBin2+ MetaBAT2	Chloroflexota	f__E44-bin15	74.72	7.59	0.46	cluster6
**LB**	LB_nanophase_bin.78	NanoPhase	Actinobacteriota	f__Anoxymicrobiaceae	50.86	1.72	0.45	cluster20
**LB**	LB.bin06	CONCOCT+ MaxBin2+ MetaBAT2	Verrucomicrobiota	o__UBA8416	63.55	3.49	0.41	cluster2
**LB**	LB_nanophase_bin.106	NanoPhase	Krumholzibacteriota	f__LZORAL124-64-63	60.21	0	0.40	cluster4
**LB**	LB.bin01	CONCOCT+ MaxBin2+ MetaBAT2	Zixibacteria	f__PGXB01	60.12	1.1	0.38	cluster12
**LB**	LB.bin04	CONCOCT+ MaxBin2+ MetaBAT2	Eisenbacteria	g__JACRPT01	55.01	1.1	0.35	cluster7
**LB**	LB.bin08	CONCOCT+ MaxBin2+ MetaBAT2	Bacteroidota	g__FEN-979	61.32	2.63	0.35	cluster3
**LB**	LB.bin09	CONCOCT+ MaxBin2+ MetaBAT2	d__Archaea; p__Iainarchaeota	g__JAAZKV01	56.63	0	0.34	cluster16
**LB**	LB.bin02	CONCOCT+ MaxBin2+ MetaBAT2	Krumholzibacteriota	f__LZORAL124-64-63	58.63	1.1	0.33	cluster4
**LB**	LB.bin10	CONCOCT+ MaxBin2+ MetaBAT2	Bacteroidota	g__JAAYVN01	58.8	3.03	0.31	cluster17
**LB**	LB.bin11	CONCOCT+ MaxBin2+ MetaBAT2	Bacteroidota	f__FEN-979	59.05	9.14	0.30	cluster5

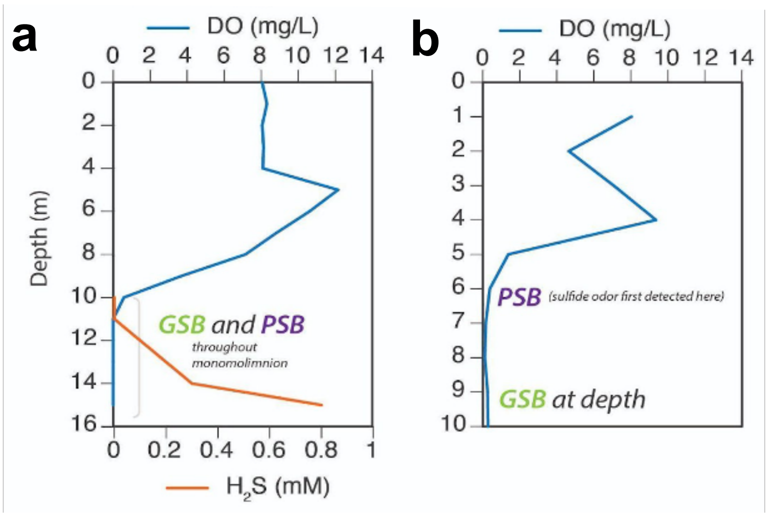

Supplementary Figure 1. Water column dissolved oxygen (DO) and sulfide (H_2S) concentrations and distribution of PSB and GSB at (a) Lime Blue and (b) Poison Lake.

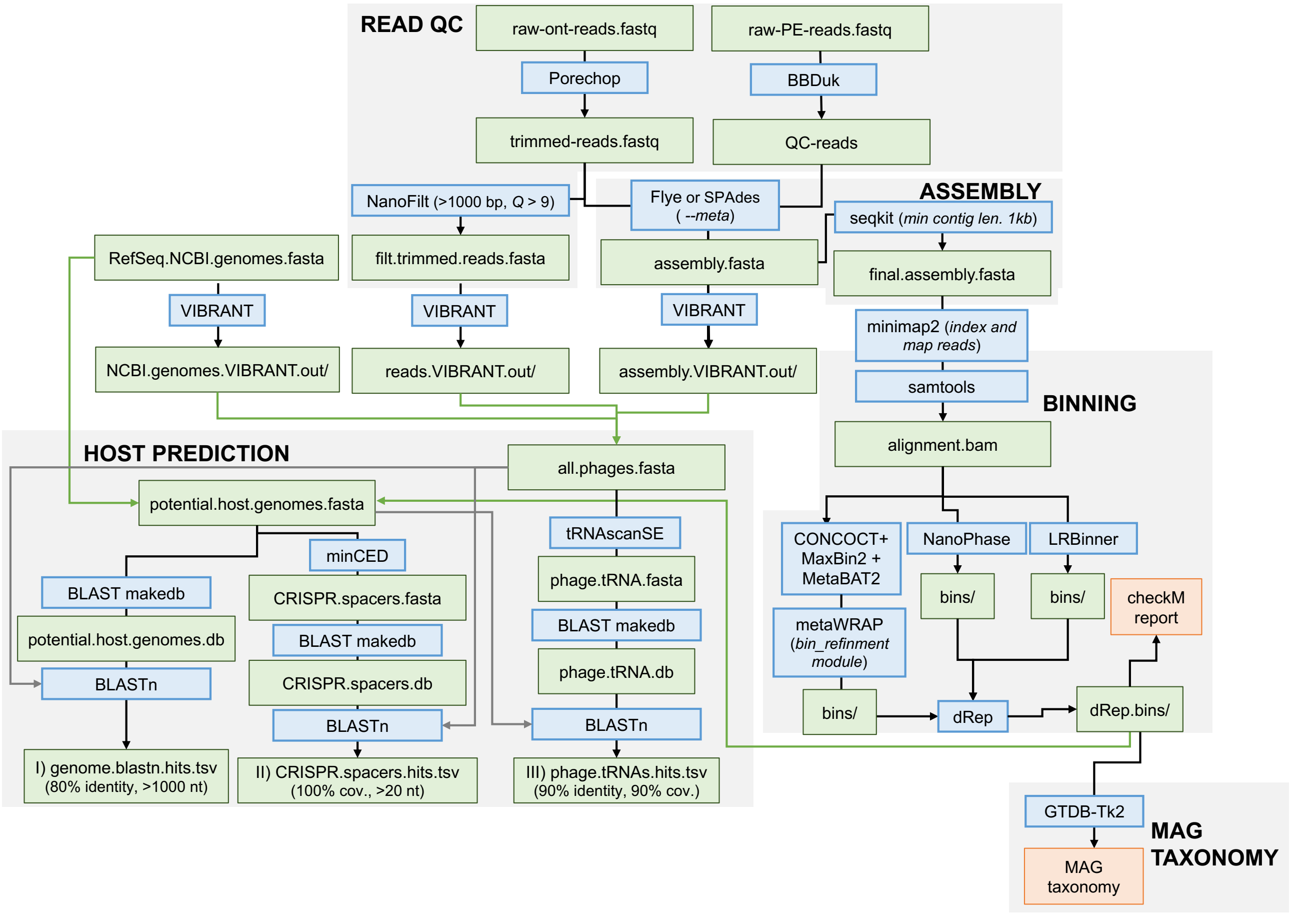

Supplementary Figure 2. Workflow outline of metagenomic analysis conducted as part of this study.

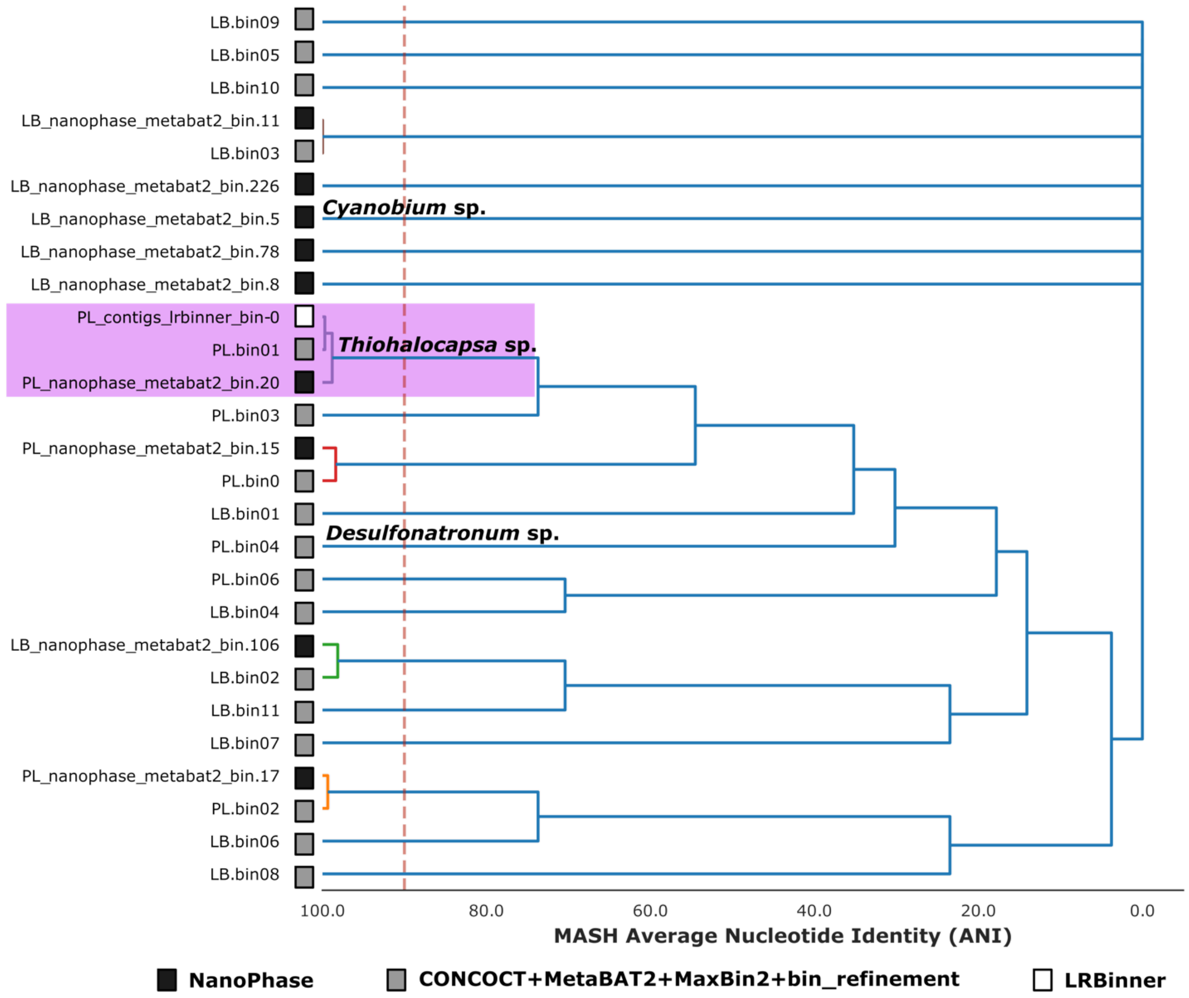

Supplementary Figure 3. MASH clustering of metagenomic bins from three binning strategies utilized in the recovery of putative host bacterial genomes and species level taxonomy predicted by GTDB-Tk2. Red dotted line denotes the 95% ANI threshold for species demarcation.

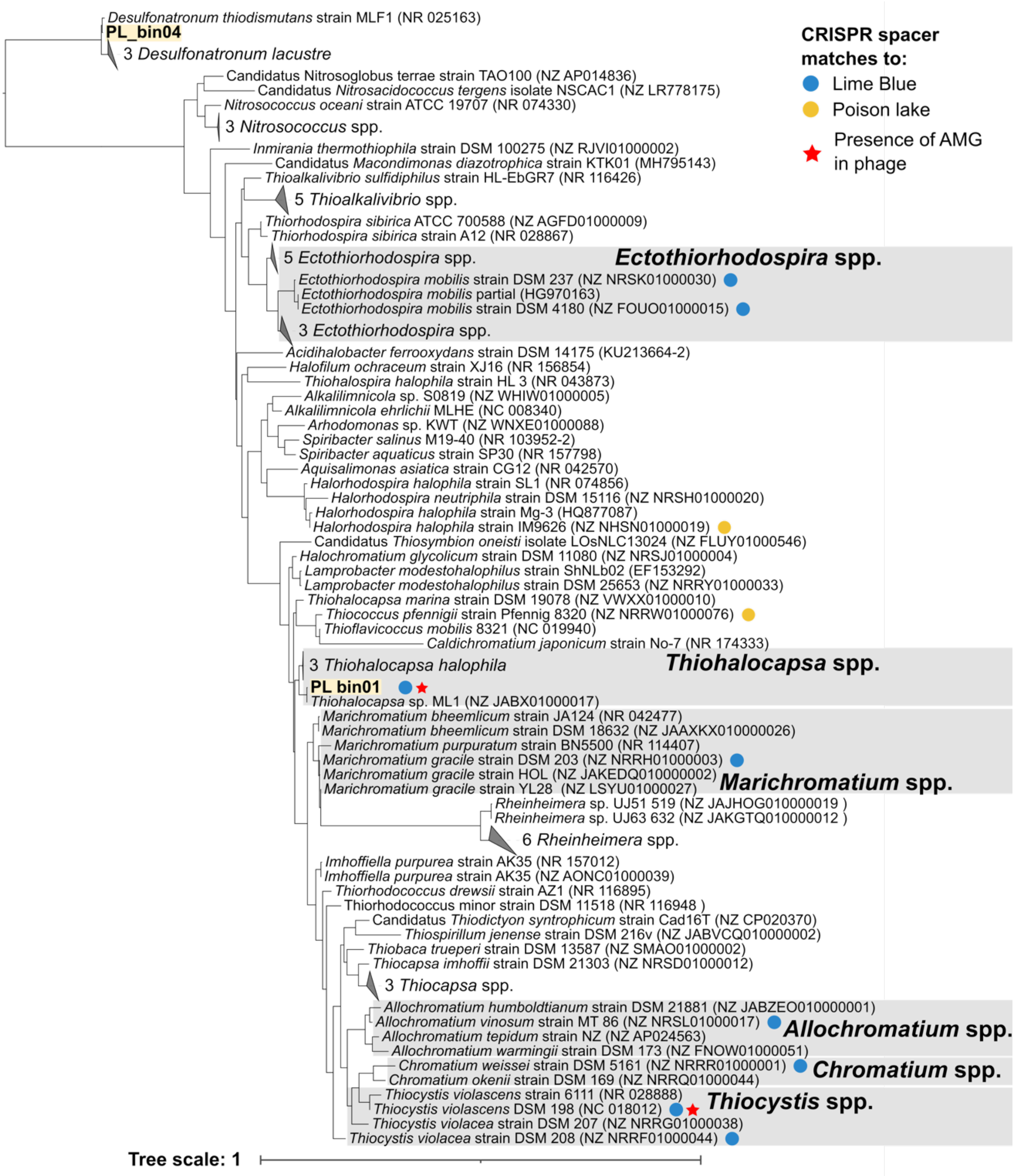

Supplementary Figure 4. Phylogenetic tree of phages, and MAG phylogeny displaying phage hosts from the PSB as predicted by CRISPR spacer matches (minimum length 20 nt; 100% identity, maximum of 2 mismatches/gaps).

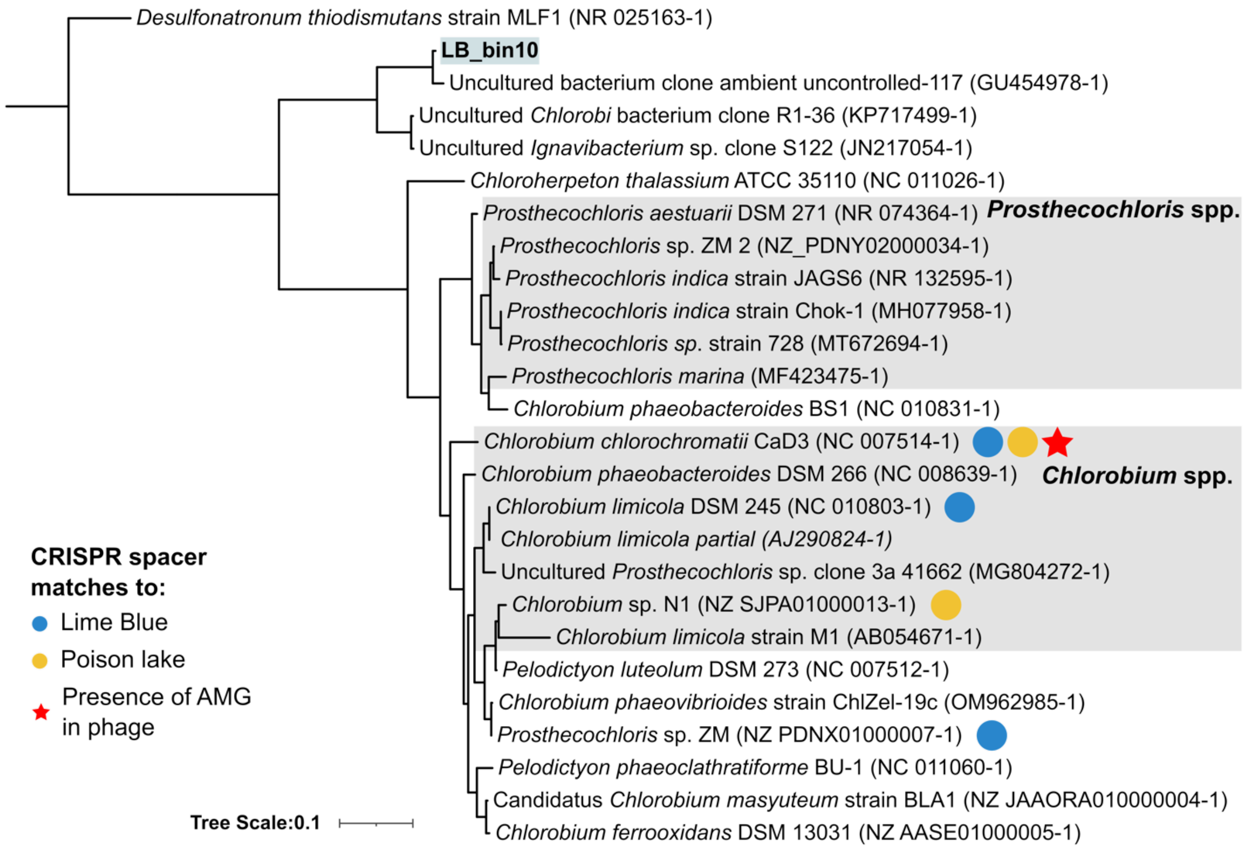

Supplementary Figure 5. Phylogenetic tree of phages, and MAG phylogeny displaying phage hosts from the GSB as predicted by CRISPR spacer matches (minimum length 20 nt; 100% identity, maximum of 2 mismatches/gaps).

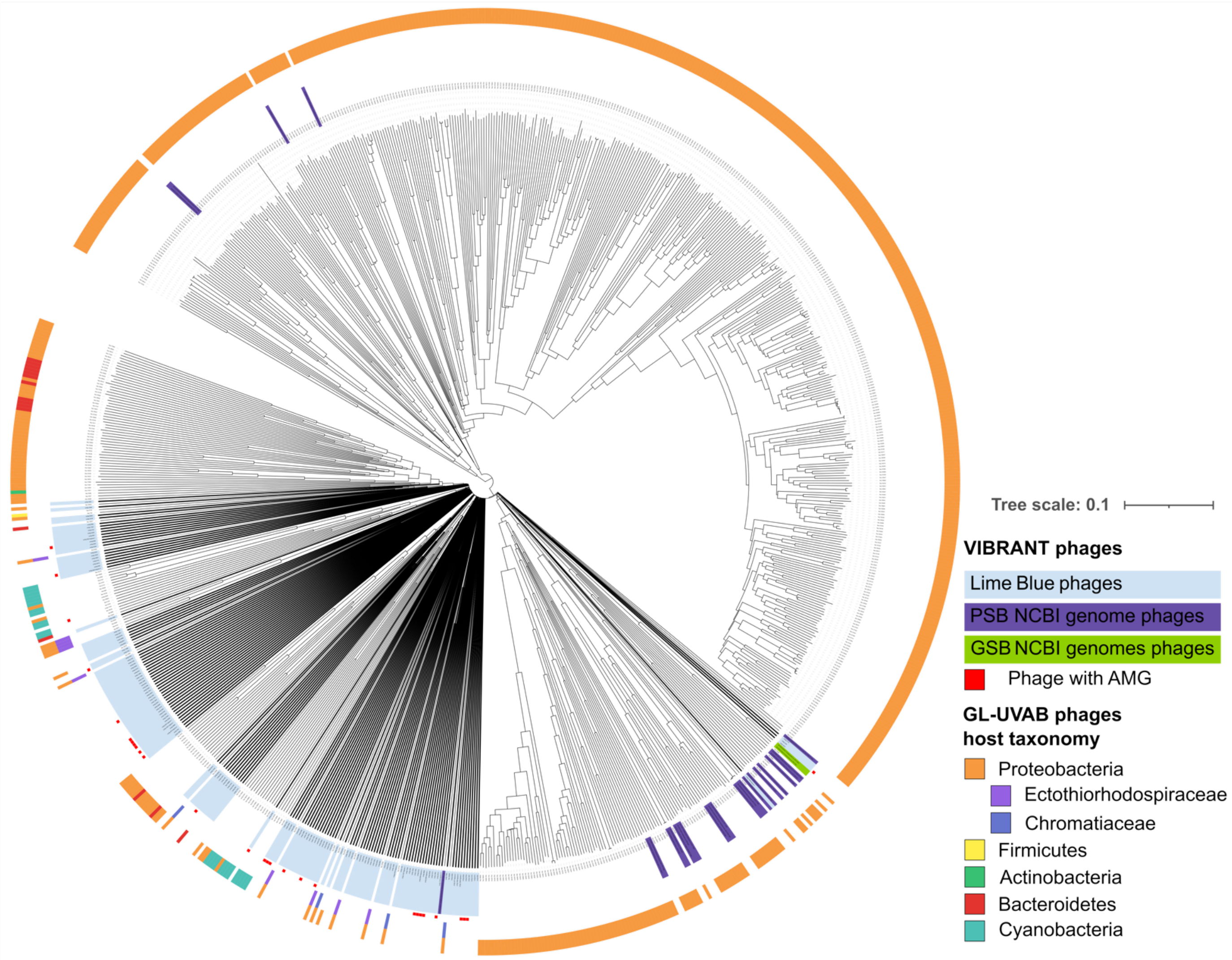

Supplementary Figure 6. Clustering of VIBRANT identified phages from Lime Blue metagenome and PSB genomes GSB genomes and the reference phage genomes based on their Dice distance with supporting branch lengths.

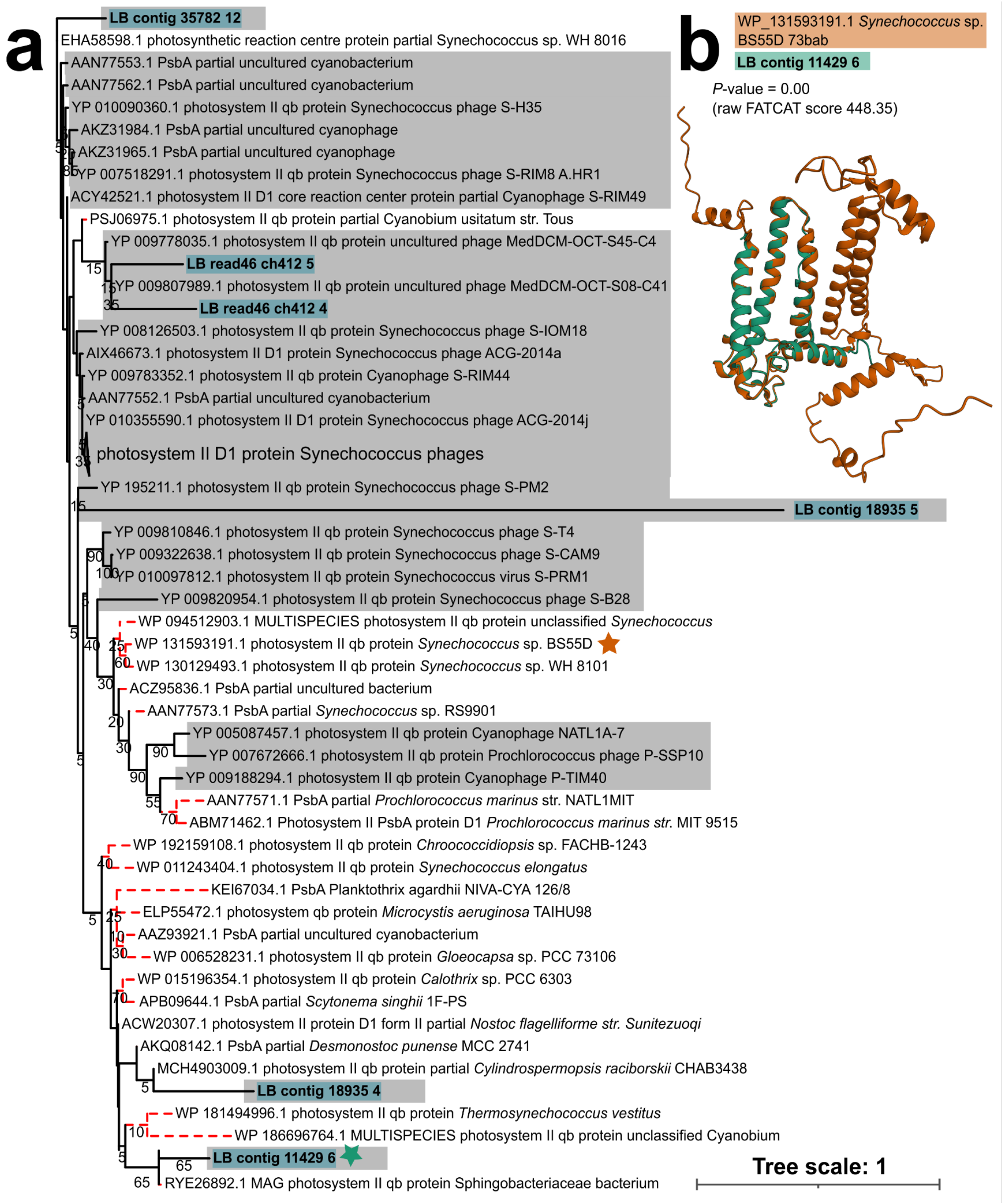

Supplementary Figure 7. Protein phylogeny and predicted structure comparison of putative phage-encoded PsbA. a) Phylogenetic analysis of 45 PsbA amino acid sequences from bacteria and phages from this study (Blue: Lime Blue phages) and RefSeq non-redundant viral and bacterial proteins. Red dotted branches indicate bacterial proteins and grey highlight viral proteins. b) The superimposed protein structure is the result of a pairwise comparison between PsbA proteins from *Synechococcus* sp. BS55D (green) and Lime Blue phage contig11429. Proteins that are folded are denoted by a star on the phylogenetic tree with the aforementioned colors, and *p*-value of the alignment and raw FATCAT score are reported within the figure.

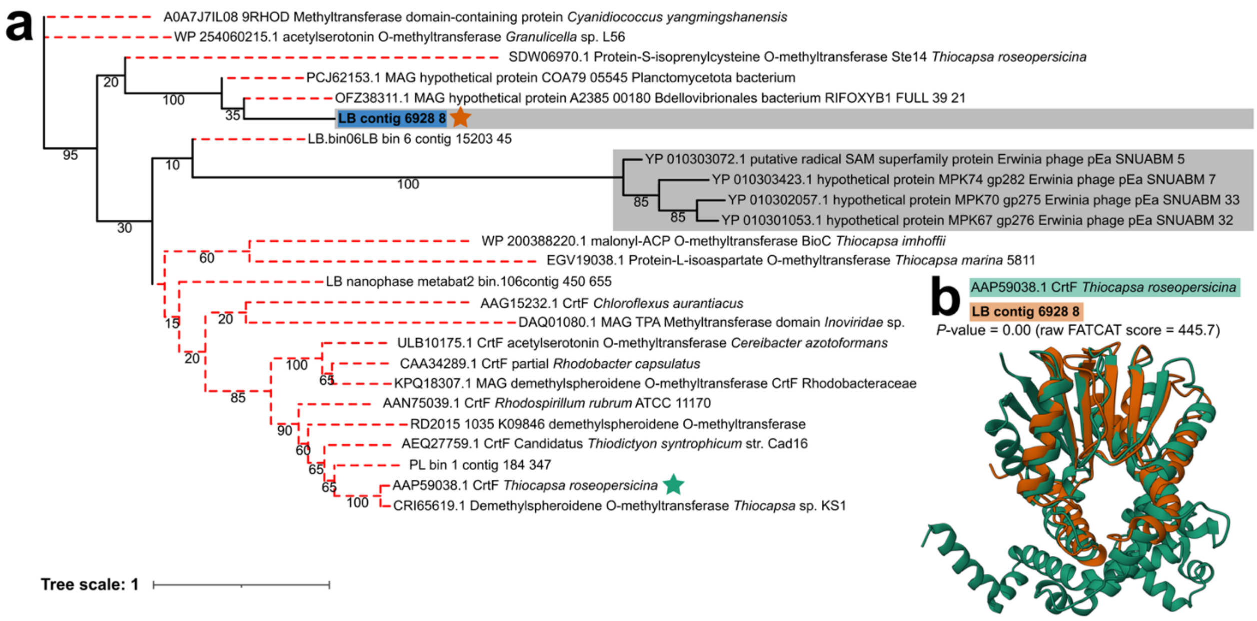

Supplementary Figure 8. Protein phylogeny and predicted structure comparison of putative phage-encoded CrtF. a) Phylogenetic analysis of 25 bacterial and viral CrtF amino acid sequences from this study (Blue: Lime Blue phages) and RefSeq non-redundant viral and bacterial proteins. Red dotted branches indicate bacterial proteins, and grey highlight viral proteins, values represent bootstrapping results. (b) Pairwise comparison of folded proteins from (green) CrtF protein encoded by *Thiocapsa reseopersicina* (AAP59038.1) and (orange) the predicted CrtF from Lime blue phage contig6928. Proteins that are folded are denoted by a star on the phylogenetic tree with the aforementioned colors, and *p*-value of the alignment and FATCAT score are reported within the figure.

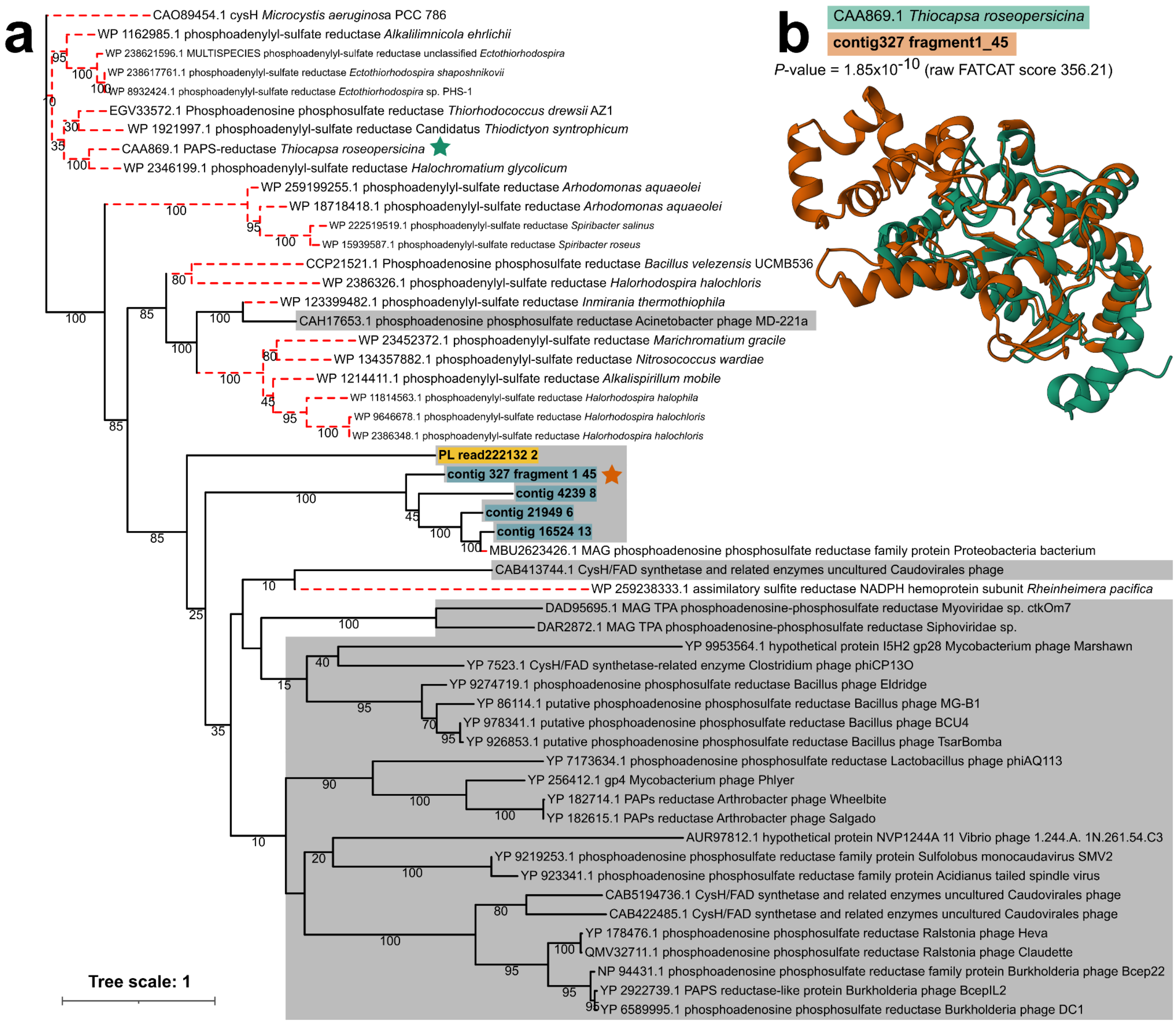

Supplementary Figure 9. Protein phylogeny and predicted structure comparison of putative phage-encoded CysH. a) Phylogenetic analysis of 53 bacterial and phage CysH amino acid sequences from this study (yellow: Poison Lake phages; Blue: Lime Blue phages) and RefSeq non-redundant viral and bacterial proteins. Red dotted branches indicate bacterial proteins, and grey highlight viral proteins. b) CysH proteins from *Thiocapsa roseopersicina* (CAA869.1) and a Poison Lake putative phage CysH. Proteins that are folded are denoted by a star on the phylogenetic tree with the aforementioned colors, and *p*-value of the alignment and FATCAT score are reported within the figure.
