# Supplementary material for: Viruses of sulfur oxidizing phototrophs encode genes for pigment, carbon, and sulfur metabolisms
Source: Commun Earth Environ. 2023 Apr 19;4(1):126. doi: 10.1038/s43247-023-00796-4 (PMC11041744; doi:10.1038/s43247-023-00796-4)
Supplement: Supplementary file 6 — Reporting Summary [file 43247_2023_796_MOESM6_ESM.pdf]

nature portfolio

Corresponding author(s): Cynthia B. Silveira

Last updated by author(s): Mar 24, 2023

Reporting Summary

Nature Portfolio wishes to improve the reproducibility of the work that we publish. This form provides structure for consistency and transparency in reporting. For further information on Nature Portfolio policies, see our Editorial Policies and the Editorial Policy Checklist.

Statistics

For all statistical analyses, confirm that the following items are present in the figure legend, table legend, main text, or Methods section.

n/a	Confirmed	
☐	☒	The exact sample size (*n*) for each experimental group/condition, given as a discrete number and unit of measurement
☐	☒	A statement on whether measurements were taken from distinct samples or whether the same sample was measured repeatedly
☐	☒	The statistical test(s) used AND whether they are one- or two-sided<br>*Only common tests should be described solely by name; describe more complex techniques in the Methods section.*
☒	☐	A description of all covariates tested
☒	☐	A description of any assumptions or corrections, such as tests of normality and adjustment for multiple comparisons
☒	☐	A full description of the statistical parameters including central tendency (e.g. means) or other basic estimates (e.g. regression coefficient) AND variation (e.g. standard deviation) or associated estimates of uncertainty (e.g. confidence intervals)
☐	☒	For null hypothesis testing, the test statistic (e.g. *F*, *t*, *r*) with confidence intervals, effect sizes, degrees of freedom and *P* value noted<br>*Give P values as exact values whenever suitable.*
☒	☐	For Bayesian analysis, information on the choice of priors and Markov chain Monte Carlo settings
☒	☐	For hierarchical and complex designs, identification of the appropriate level for tests and full reporting of outcomes
☒	☐	Estimates of effect sizes (e.g. Cohen's *d*, Pearson's *r*), indicating how they were calculated

Our web collection on statistics for biologists contains articles on many of the points above.

Software and code

Policy information about availability of computer code

Data collection: No computer code was used for data collection.

Data analysis: Computer codes used for analyses are publicly available in the repository Figshare and described in the Availability Statement.

For manuscripts utilizing custom algorithms or software that are central to the research but not yet described in published literature, software must be made available to editors and reviewers. We strongly encourage code deposition in a community repository (e.g. GitHub). See the Nature Portfolio guidelines for submitting code & software for further information.

Data

Policy information about availability of data

All manuscripts must include a data availability statement. This statement should provide the following information, where applicable:

- Accession codes, unique identifiers, or web links for publicly available datasets
- A description of any restrictions on data availability
- For clinical datasets or third party data, please ensure that the statement adheres to our policy

The Nanopore metagenomic sequencing data generated here are available in the Sequence Reads Archives (SRA) repository under the BioProject PRJNA842402: Lime Blue sediment (SRS13178833) and Poison Lake water (SRS13178834). CSV files are available on Figshare (DOIs in the Data Availability Satatement)

Human research participants

Policy information about studies involving human research participants and Sex and Gender in Research.

Reporting on sex and gender	The study did no involve any human research.
Population characteristics	See above.
Recruitment	See above.
Ethics oversight	See above.

Note that full information on the approval of the study protocol must also be provided in the manuscript.

Field-specific reporting

Please select the one below that is the best fit for your research. If you are not sure, read the appropriate sections before making your selection.

☐ Life sciences ☐ Behavioural & social sciences ☒ Ecological, evolutionary & environmental sciences

For a reference copy of the document with all sections, see nature.com/documents/nr-reporting-summary-flat.pdf

Ecological, evolutionary & environmental sciences study design

All studies must disclose on these points even when the disclosure is negative.

Study description	Genomic study.
Research sample	Sediment and water from two anoxic lakes.
Sampling strategy	One representative sample was taken from each lake for a first description of their viral communities.
Data collection	Water was sampled with a Niskin bottle and sediments with a freeze-core.
Timing and spatial scale	Only one time point was collected for each lake.
Data exclusions	No data were excluded.
Reproducibility	The data was compared with genomes publicly available in the National Center for Biotechnology Information.
Randomization	As only one sample was collected from each lake, randomization was not applicable.
Blinding	Blinding was not possible due to the small sample size.

Did the study involve field work? ☒ Yes ☐ No

Field work, collection and transport

Field conditions	Samples were collected in the summer, no atypical rainfall or weather occurred.
Location	Eastern Washington, U.S. (48N, 119W)
Access & import/export	Sampling permits are not required for the two locations. The lakes are located in a tourist-accessible area.
Disturbance	Disturbance was minimal due to small sample size.

Reporting for specific materials, systems and methods

We require information from authors about some types of materials, experimental systems and methods used in many studies. Here, indicate whether each material, system or method listed is relevant to your study. If you are not sure if a list item applies to your research, read the appropriate section before selecting a response.

Materials & experimental systems

n/a	Involved in the study
☒	☐ Antibodies
☒	☐ Eukaryotic cell lines
☒	☐ Palaeontology and archaeology
☒	☐ Animals and other organisms
☒	☐ Clinical data
☒	☐ Dual use research of concern

Methods

n/a	Involved in the study
☒	☐ ChIP-seq
☒	☐ Flow cytometry
☒	☐ MRI-based neuroimaging
